# COVID-19 Amongst Travelers at Points of Entry in Nepal: Screening, Testing, Diagnosis and Isolation Practices

**DOI:** 10.3390/tropicalmed7060099

**Published:** 2022-06-10

**Authors:** Koshal Chandra Subedee, Krishna Prasad Paudel, Mohammed Khogali, Amrit Pokhrel, Palanivel Chinnakali, Nishant Thakur, Deepak Timsina, Rabin Gautam, Anisur Rahman, Shrawan Kumar Mandal, Mahendra Dhose Adhikari, Anthony D. Harries

**Affiliations:** 1 Department of Health Services, Epidemiology and Disease Control Division, Kathmandu 44600, Nepal; pokhrelashu@gmail.com (A.P.); neeshant14@gmail.com (N.T.); 2 Ministry of Health and Population, Kathmandu 44600, Nepal; kpkalyan@gmail.com; 3 Special Programme for Research and Training in Tropical Diseases (TDR), World Health Organization (WHO), 1211 Geneva, Switzerland; khogalim@who.int; 4 Department of Preventive and Social Medicine, Jawaharlal Institute of Post Graduate Medical Education and Research, Puducherry 605006, India; palaniccm@gmail.com; 5 Abt Associates Inc., Kathmandu 44600, Nepal; deepak_timsina@ssbhnepal.org; 6 World Health Organization (WHO), Country Office, Kathmandu 44600, Nepal; rgautam@who.int; 7 World Health Organization (WHO), Country Office, New Delhi 110029, India; rahmanan@who.int; 8 Sukraraj Tropical and Infectious Disease Hospital, Kathmandu 44600, Nepal; fusion7722@gmail.com; 9 District Health Office, Nuwakot 44900, Nepal; mahendradhose@gmail.com; 10 International Union Against Tuberculosis and Lung Disease (The Union), 2 Rue Jean Lantier, 75001 Paris, France; adharries@theunion.org; 11 Department of Clinical Research, Faculty of Infectious and Tropical Diseases, London School of Hygiene and Tropical Medicine, Keppel Street, London WC1E 7HT, UK

**Keywords:** screening for COVID-19, Nepal, travelers, points of entry, border crossings, COVID-19 isolation procedures, rapid antigen diagnostic test, lateral flow antigen test, SORT IT, operational research

## Abstract

WHO recommends surveillance for COVID-19 among travelers at Points of Entry (POE) to countries. At 13 selected POE at the Nepal-India border, between March 2021 and July 2021, we describe the screening, testing, diagnosis and isolation practices of COVID-19 amongst travelers. Those who stayed in India or elsewhere for > one day and those who did not have a negative RT-PCR result within the last 72 h of travel were tested for COVID-19 with rapid antigen diagnostic tests. Daily surveillance reports maintained at POE were used for analysis. Of 337,338 travelers screened, 69,886 (21%) were tested and 3907 (6%) were diagnosed with COVID-19. The proportions tested averaged 15% during April-May when screened numbers were high and increased to 35% in July when screened numbers had decreased. The proportions diagnosed positive peaked at 10% in April-May, but decreased to below 1% in June and July. Testing coverage varied from 0–99% in the different POE. Most COVID-19 cases were Nepalese, male, <60 years of age, migrant workers and presented with fever. Of COVID-19 cases, 32% had home-based isolation, 64% underwent community-based isolation and the remainder either went to hospital or returned to India. In conclusion, about one fifth of travelers overall were tested, with coverage varying considerably over time and among different POE. Strengthening surveillance processes at POE is needed.

## 1. Introduction

Coronavirus disease 2019 (COVID-19) is an infectious disease caused by a novel coronavirus called severe acute respiratory syndrome coronavirus 2 (SARS-CoV-2). Since the identification of the first case of COVID-19 in Wuhan city, Hubei province, China, in late 2019, cases have spread rapidly throughout the world. On 11 March 2020, the World Health Organization (WHO) declared COVID-19 to be a global pandemic. As of mid-March 2022, there were 455 million COVID-19 cases globally reported to the WHO along with over 6 million deaths [1].

One of the reasons for the rapid spread of the COVID-19 cases across the world is population movement. This includes infected people travelling and spreading infection, and this facilitates the transmission of SARS-CoV-2 from countries with an outbreak of COVID-19 to countries that are COVID-19 naïve [2,3]. Given the potential for rapid and exponential spread of COVID-19, new cases should be identified, reported and isolated as quickly as possible. In this light, the WHO has recommended a combination of surveillance strategies for early detection and reporting of COVID-19 cases, including surveillance for COVID-19 among travelers at Points of Entry (POE). Several countries have been implementing screening and testing measures at POE, which include sea ports, airports and ground crossings [2,3]. These measures are linked to the isolation, quarantine and management of COVID-19 infected travelers [2], which reduce the risk of importing new variants of SARS-CoV-2 and prevent further spread of COVID-19 in the community [4].

One of the methods being used for testing travelers at POE is the lateral flow antigen (LFA) test. Although the gold standard for the diagnosis of SARS-CoV-2 is detection of viral RNA through nucleic acid amplification and real time polymerase chain reaction (RT-PCR), the LFA test is a reasonable alternative because it is inexpensive, widely available, easy to use and provides results in 15-30 min [5]. The LFA test has high specificity, approaching 100% in some studies, but sensitivity is much lower and crucially dependent on viral load [6,7]. While cases can therefore be missed, this does mean, however, that the LFA test is likely to identify people with COVID-19 who have high viral loads, who, in turn, are those most likely to transmit infection to others.

In Nepal, the first case of COVID-19 was detected in January 2020: this was a student who had a travel history to Wuhan city in China [8]. Since then, the number of cases has increased to reach a total of nearly 980,000 reported cases by March 2022 [9]. The first wave of cases occurred between 23 January 2020 and 14 March 2021, and reached 275,231, while the second wave of cases occurred between 15 March 2021, and December 31, 2021, and reached 649,402 [10,11]. During the early phase of the pandemic, the majority of COVID-19 cases were imported from the neighboring country India, due to the daily influx of travelers crossing over at the Indo-Nepal borders [12]. As a result of this, and in line with International Health Regulations and WHO recommendations, Nepal established a regular surveillance system in selected POE between Nepal and India in mid-March 2021. The number of POE undertaking surveillance of travelers from India to Nepal was then expanded to 13, based on a government cabinet decision. According to the national surveillance protocol, travelers who were in India, or another country, for more than one day, and travelers with no negative RT-PCR result within the last 72 h of travel, and those presenting with fever and other COVID-19 symptoms, needed to be screened for COVID-19 and tested with an LFA test.

Data on screening, testing and positive LFA results at each POE were collected and collated on a daily basis, with findings reported to the Epidemiology and Disease Control Division (EDCD) of the Ministry of Health and Population (MoHP) in Nepal. To date, there has been no formal analysis of these data and there is no information about how well the surveillance system and isolation procedures for COVID-19 cases have been performing. Daily testing is vitally dependent on the availability of LFA tests, and if these are in short supply, daily testing numbers will reduce. Information, therefore, on performance at each POE is important for tackling and controlling the COVID-19 pandemic. It will also provide valuable data for the procurement and distribution of LFA tests, as well as for the deployment of adequate human resources at these busy POE. We need to know whether the variables in the monitoring database are adequate to provide the necessary information about who needs testing and who is getting COVID-19. For those with COVID-19, we also need to know where the patients are being isolated and, if the database allows, the clinical progress of these patients.

We, therefore, set up this study to report on the screening, testing, diagnosis and isolation practices of COVID-19 at 13 designated ground crossing POE in Nepal. Among travelers to Nepal at these selected ground-crossing POE between 19 March 2021 and 15 July 2021 (17 week period), specific objectives were to document: (i) overall, and for each POE, the number of screened travelers and proportions tested and diagnosed with COVID-19, (ii) the trends in travelers screened, tested and diagnosed with COVID-19 for all POE per week, (iii) the socio-demographic characteristics and clinical presentation of persons tested positive for COVID-19 through LFA test, and (iv) the isolation procedures (home based, community based and hospital-based) undertaken for persons diagnosed positive for COVID-19.

## 2. Materials and Methods

### 2.1. Study Design

This was a descriptive study involving analysis of routinely collected data.

### 2.2. Setting

#### 2.2.1. General Setting

Nepal is a landlocked country in South Asia with a population of 2,64,94,504 [13]. It has three different geographic terrains (Mountain, Hill and Terai [low land]) and borders China to the North and India to the south, east and west. There is an active daily exchange of travelers between Nepal and India, part of which is accounted for by migrant workers travelling from Nepal to India for work.

The Ministry of Health and Population of Nepal has responded to the COVID-19 pandemic by: implementing surveillance strategies, including testing, case investigation and contact tracing along with quarantine and isolation practices; strengthening the country’s laboratory capacities; instituting infection prevention and control (IPC), case management and risk communication and community engagement (RCCE); and setting various standard public health and social measures (PHSMs) to stop the spread of the virus in line with standard operating procedures (SOPs). According to the national testing strategy for COVID-19, all patients presenting with COVID-19 symptoms and contacts identified through contact tracing must be tested using either a RT-PCR or a LFA, depending on the availability of each of these tests.

#### 2.2.2. Specific Setting (POE)

**Figure 1:**
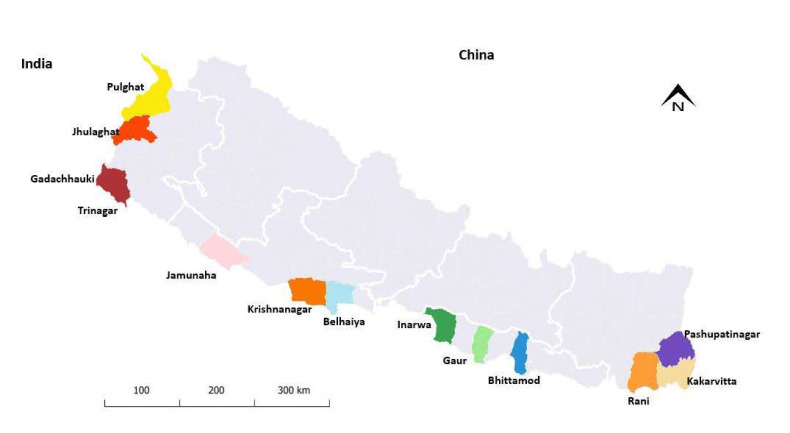
Map of Nepal showing ground crossing points of entry (POE) between Nepal and India.

There are 20 designated POE at the border between Nepal and India [14]. The surveillance mechanism for COVID-19 was established by the Ministry of Health and Population at 13 POE. Two POE are located in the western border and eastern border, and the remaining POE are located in the southern border. Initially, the surveillance activities started in two POE on 13 March 2021, and this was gradually scaled up to all the 13 POE by the end of March 2021 (Figure 1).

According to the national surveillance protocol, the type of health worker cadre carrying out the surveillance activities should include medical officers, nurses, paramedical officers and laboratory personnel. However, these differ across the different POE depending on the availability of human resources and the number of travelers crossing the border daily. Among these health workers, medical officers, paramedical officers and nurses carry out screening and management of positive cases, while laboratory staff are mainly involved in testing and diagnosis of COVID-19. Surveillance data from each POE were routinely collected, collated and reported to the EDCD on a daily basis in a structured reporting format. The reported data included aggregated information on the daily number of travelers screened, tested and diagnosed with COVID-19. Individual level data on positive cases were also reported.

#### 2.2.3. Lateral Flow Antigen (LFA) Test

The approved rapid antigen diagnostic test used in Nepal is the protein-based LFA test. A nasopharyngeal/oropharyngeal swab is collected from the posterior pharynx and tonsillar areas using a mini-tip swab with a flexible shaft. The test uses a lateral flow assay principle, and the result can be obtained after 15–30 min, based on the manufacturing type.

LFA test kits are mainly supplied by the management division of the Department of Health Services to provincial offices, which, in turn, supply the test kits to the district health offices. From district health offices, the test kits are then distributed to POE. In addition, district health offices and local municipality offices can also purchase and supply test kits to the POE. Only those LFA test kits which are approved and listed by the national public health laboratory can be procured [15]. The LFA test is offered free of charge for all travelers. However, there is no system in place to ensure regular and adequate supply of the test kits to the POE

#### 2.2.4. Screening and Testing Procedure and Practice

According to the national surveillance protocol, travelers who were in India for more than one day, and those who did not have a negative RT-PCR result within the last 72 h of travel, and those presenting with fever and other COVID-19 symptoms should be screened for COVID-19 and tested with an LFA test. The screening process consists of two parts. The first part involves collecting demographic and clinical information about the traveler, which includes name, age, sex, occupation, presence of COVID-19 symptoms (fever, cough, difficulties in breathing and other COVID-19 related symptoms, such as weakness, headache, sore throat etc.), comorbidities (hypertension, heart disease, renal disease, diabetes mellitus, chronic obstructive pulmonary disease (COPD)) and COVID-19 vaccination status. The second part is screening for fever using an infrared thermometer. All travelers who are screened, regardless of results, should then be tested for COVID-19 using the LFA test. However, if there are interruptions in the supply of the LFA tests, only travelers with fever are tested for COVID-19. Travelers with a negative LFA test, but symptoms suggestive of COVID-19, are referred for a RT-PCR test.

#### 2.2.5. Isolation Procedure of Positive COVID-19 Patients

Depending on the severity of the symptoms, patients are isolated at home, in community-based isolation centers or in hospitals. Asymptomatic patients are normally isolated at home. However, asymptomatic patients with no suitable place for home isolation are isolated in one of the community-based isolation centers. Mild cases are isolated in the community-based isolation centers and are monitored by health workers. Mild cases can also be isolated at home but are obliged to follow certain health standards as per the COVID-19 isolation guidelines [16]. Moderate and severe cases are isolated at hospital where advanced care may be needed. A decision has been made by the government to establish a multifunctional holding center with the capacity to temporarily accommodate up to 1000 COVID-19 patients while they are waiting to be transferred to different isolation centers. However, at the time of the current study, this holding center had not yet been established on the ground. Currently, patients who are awaiting transfer to isolation centers stay in tents near the POE.

### 2.3. Study Population and Time Period

The study population included all travelers that were screened for COVID-19 between 19 March 2021 and 15 July 2021 at 13 designated POE at borders between Nepal and India. These POE are shown in Figure 1. The reasons for choosing this time period included the advent of the Delta variant of SARS-CoV-2 in the country, which occurred during these months, and the end of the fiscal year in the Nepali calendar.

### 2.4. Data Variables, Data Sources, Data Collection and Validation

Data variables included: name of the POE; total and weekly numbers of travelers screened, tested and diagnosed with COVID-19 at each POE; of persons diagnosed with COVID-19, their age, sex, occupation, nationality, symptoms (including cough, sore throat and fever) and site of isolation (home-based, community-based and hospital-based). The operational definition of a screened traveler included any person who was in India for more than one day and who did not have a negative RT-PCR result within the last 72 h of travel, or any person with a documented fever during screening.

Data were recorded daily in a paper-based format and entered into an electronic database in Excel. These daily surveillance reports were sent from the POE to the surveillance section of the EDCD of the MoHP. These data were cross checked and double entered, by two independent encoders, into a data entry file which was created using Microsoft Excel. The two data files were then compared, and discordances were resolved by cross-checking with the original reports. Aggregate data were used for the first two study objectives, while individual level data were used for the last two study objectives.

### 2.5. Analysis and Statistics

Data were analyzed using EpiData analysis software version 2.2 (EpiData Association, Odense, Denmark). A descriptive analysis was undertaken using frequencies and proportions to describe travelers screened, tested and diagnosed with COVID-19. Numbers and proportions were calculated to describe the socio-demographic and clinical characteristics of patients, as well as isolation procedures.

## 3. Results

### 3.1. Travelers Screened, Tested and Diagnosed at POE during the Study Period

**Figure 2:**
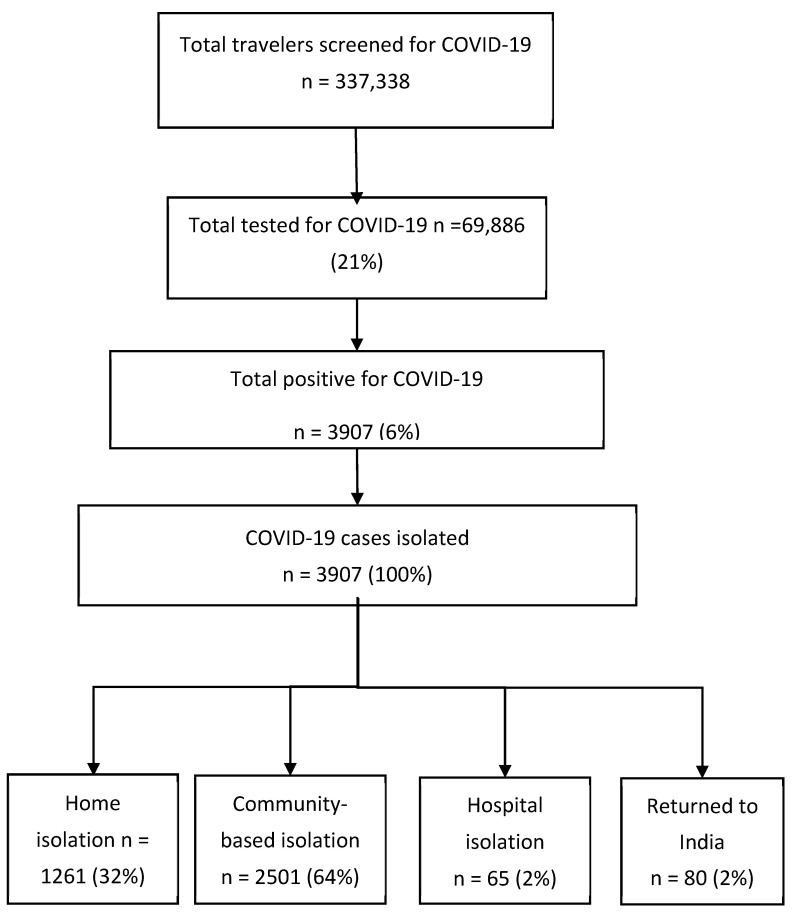
Numbers of travelers screened, tested and diagnosed with COVID-19 along with types of isolation for the management of COVID-19 at 13 POE in Nepal: 19 March 2021–15 July 2021. COVID-19 = Coronavirus Disease-19; POE = Points of Entry.

The number of travelers screened, tested and diagnosed positive for COVID-19 over the study period at the 13 POE are shown in the flow diagram in Figure 2. Of the 337,338 screened travelers, 69,886 (21%) were tested for COVID-19 and 3907 (6%) were positive.

The numbers screened, tested and diagnosed positive at each POE are shown in Table 1. Over the whole study period, there was one POE (Jamunaha) that screened over 130,000 travelers, there was one (Trinagar) that screened nearly 67,000, there were five (Belahiya, Gaddachauki, Inarwa, Kakarbhitta and Krishnanagar) that screened between 10,000-40,000 and the remaining POE screened less than 10,000. The three POE (Jamunaha, Trinagar and Gaddachauki) with the highest number of screened travelers, altogether tested 53,070 (75.9%) of the total of 69,886 travelers who were tested. In Bhittamod POE no traveler was tested; in the remaining POE, the proportions tested ranged from 4.8% to 98.8%. The testing rates in the three POE with highest numbers screened were: Jamunaha 7.3%, Trinagar 41.6% and Gaddachauki 39.5%.

The highest test positivity rate was observed in Krishnanagar POE (13.5%) followed by 10.1% in Inarwa POE and 8.9% each in Rani and Trinagar POE. In the other POE, test positivity rates varied from 0.2% to 5.9%.

### 3.2. Trends in Screening, Testing and Diagnosis at POE during the Study Period

**Figure 3:**
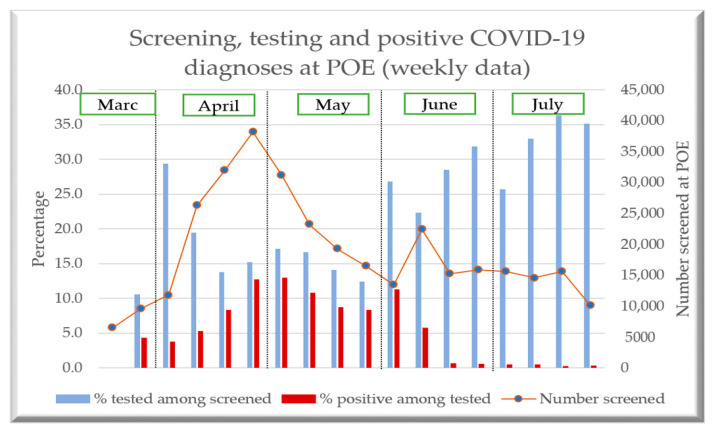
Screening, testing and positive COVID-19 diagnoses in travelers by week at all POE, Nepal: 19 March 2021–15 July 2021. COVID-19= Corona Virus Disease-19; POE = Points of Entry.

Weekly trends in the proportions of travelers screened, tested and diagnosed over the study period are shown in Figure 3. The peak in numbers screened occurred in the weeks of April, with a gradual decline in May followed by a small increase and plateau in June and July. The proportions tested were about 15% during April and May and then increased to 25% in June and 35% in July. The numbers and proportions diagnosed positive reached 10% in April and into May, and thereafter dropped to below 1% in June and July.

### 3.3. Characteristics of Travelers Testing Positive for COVID-19

Socio-demographic and clinical characteristics of travelers testing positive for COVID-19 are shown in Table 2. The commonest age group was between 15–44 years, constituting 82% of all travelers, with those aged 15–29 years constituting over half of the total number. There were significantly more males than females. Over 80% of travelers were migrant workers and over 95% were Nepalese. The commonest symptom was fever, reported in nearly 95% of all the travelers.

### 3.4. Types of Isolation for Travelers with COVID-19

The different types of isolation for travelers diagnosed with COVID-19 are shown in Table 3. About one third underwent home-based isolation and just under two thirds underwent community-based isolation. A few travelers (mainly those entering at Jamunaha POE) underwent hospital-based isolation, while 80 in total (and all from Inarwa) returned to India.

## 4. Discussion

This is the first published study from Nepal describing the process and results of screening, testing, diagnosis and isolation of COVID-19 cases at 13 land border crossings in the country. There were four key findings.

First, the proportion of travelers tested ranged from 12% to 29% during April and May, when the number of travelers screened was highest. However, the proportion tested increased during June and July to between 22% to 36%, when the number of travelers screened was low. During the last week of April and the first week of May, large proportions of those tested were diagnosed positive with COVID-19. This contrasted with June and July when only a small proportion of those tested were diagnosed. The reasons for these differences are unclear, but the lower proportions of travelers tested in April and May could have been due to shortages of staff at POE, training issues, staff fatigue, poor communication across stakeholders and supply issues with rapid antigen diagnostic kits: all of these have been reported as challenges at POE in other countries [17,18,19].

Second, there was a huge variation in numbers screened and proportions tested and diagnosed across the 13 POE. Overall, one out of five travelers were tested. However, the proportions of travelers tested at the different POE ranged from 0 to almost 100%, and these results seemed to have little association with the numbers of travelers being screened. Nevertheless, at the POE (Jamunaha) where the numbers screened were highest (~0.1 million), the testing proportion was only 7%. Our study was not set up to explore reasons for these findings. Conventionally, many land border crossings do not have sufficient health care staff as the checks at border crossings are often focused on screening for narcotics and other illegal importations [17]. With the emergence of COVID-19 in January 2020, many of the POEs were strengthened by the repurposing and posting of health care staff from other health facilities as a makeshift arrangement, but whether these postings were retained into the pandemic needs to be clarified. It is likely that the health care manpower in the districts where the POE were located, and the stage of pandemic (COVID-19 case load) in the community, had an important effect on the manpower and rapid diagnostic test kit availability at the POE.

Third, the majority of the travelers diagnosed with COVID-19 were Nepalese, male and less than 60 years of age and four out of five were migrant workers. Information on the demographic characteristics of all travelers being screened would have been helpful. However, since the onset of the pandemic, international travel by high-risk groups, like the elderly, has become increasingly difficult with entry restrictions and mandatory quarantines [20] and this would likely have caused a reduction in the number of elderly travelers in our setting.

Fourth, most of those diagnosed with COVID-19 underwent either home- based isolation (one third) or were placed in community-based isolation centers (two thirds), presumably with asymptomatic or mild illness. This pattern of isolation aligns with the age distribution of the travelers diagnosed with COVID-19, of whom the majority were aged less than 60 years. It is well established that serious morbidity and mortality from COVID-19 increase with increasing age, particularly from the age of 60 years or more [21,22,23]. Since most of those diagnosed with COVID-19 were migrant workers, it is possible their housing conditions (number of rooms, family members and poor ventilation) might not have met the criteria for home isolation and some of the migrants’ homes might also have been far away from the POE. In these cases, the migrants might have been placed into a community-based isolation center, even though they had asymptomatic infection. One contrasting finding was that in one POE (Jamunaha), where screening numbers were the highest, 14% of travelers with COVID-19 were hospitalized. It is unclear here whether testing was offered only to travelers with severe symptoms, or the availability of a nearby hospital led to higher hospital admissions at this particular POE.

The strengths of this study were the inclusion of 13 major POE in Nepal out of a total of 20 at the Nepal-India border and the availability of data on management practices after COVID-19 had been diagnosed. The conduct and reporting of the study were also in accordance with the STROBE guidelines (Strengthening the Reporting of Observational Studies in Epidemiology) statement [24].

However, there were some limitations. First, data on the number of travelers eligible for testing, as well as the testing criteria used for each traveler, were not available and the lack of this information made it difficult to compare the proportions tested across POE. Second, it would have been helpful to have information on the number and type of manpower at POE, and their training, as well as information on stock outs of testing kits to allow a better understanding about why testing coverage varied over time and between different POE. Third, we did not have information on the clinical outcome of the travelers diagnosed with COVID-19 during isolation. Fourth, the sensitivity of LFA tests was low compared to RT-PCR based tests and information on RT-PCR tests done along with the test results in those travelers with symptoms, but who were LFA negative, was not available. These data would have been helpful to assess the need for repeat RT-PCR tests at border crossings so that supplies could be matched with demand and stock-outs thereby avoided.

Despite these limitations, this study has two important operational implications. First, our study findings strongly suggest the need to strengthen the process of epidemiological surveillance at POE. The reasons for huge variations across POE in the numbers and proportions of travelers tested for COVID-19 need to be investigated and better understood. This can probably be achieved in several ways. There is a need to have standard eligibility criteria for testing. Only those with symptoms and signs suggestive of COVID 19 infection on screening should be tested for COVID-19. This would help to decrease the workload for understaffed POE and allow testing kits to be used only when is necessary. There is a need to expand the number of variables included in the monitoring process, such as data on symptoms (presence or absence of fever), who had a negative RT-PCR and clinical progress of COVID-19 cases sent for different types of isolation. It would also help to change over to real-time digital recording and reporting. The latter might contribute to improving distribution and supply of kits to POE where they are most needed. Of note, the move to a digital and real time monitoring process would require financial support. Strong monitoring processes with periodic assessment and supervision of manpower and training are also necessary for establishing a good surveillance network at POE to combat future infectious disease transmission across the borders. Calls for better algorithms, decision support mechanisms and financing for improved entry and exit into countries have been made, and these calls support our recommendations for strengthened surveillance at border crossings in Nepal [25,26,27].

Second, although the majority of COVID-19 cases were managed at home or in community isolation centers, it is possible that some needed onward referral to hospitals because of deterioration in clinical illness. Better ways of monitoring the clinical progress of patients who need referral to higher levels of care are needed. Feedback mechanisms should be established between POE and home-based isolation, community-based isolation centers, health facilities and hospitals, and again this can only be realistically done with digital systems.

## 5. Conclusions

Using routine data from 13 POE in Nepal during March 2021 to July 2021, we were able to describe the total number of travelers screened, the proportions tested and diagnosed with COVID-19, as well as their demographic profiles and management. Testing coverage varied considerably over time and between POE. Most travelers diagnosed with COVID-19 were Nepalese, male, aged less than 60 years and migrant workers, and, of those diagnosed with COVID-19, only a few required hospital admission and care. Strengthening and periodically assessing the performance of screening processes at POE will help to improve infectious disease surveillance and build better health system resilience at border crossings in Nepal.

## Figures and Tables

**Table 1:** Screening, testing and positive COVID-19 diagnoses in travelers at each POE in Nepal: 19 March 2021–15 July 2021.

Designated POE	Screened *n*	Tested *n* (%)		COVID-19 Positive *n* (%)	
Total	337,338	69,886	(21)	3907	(6)
Belahiya	25,595	5467	(19)	124	(2)
Gaddachauki	39,571	15,611	(40)	116	(1)
Gaur	3385	602	(18)	13	(2)
Inarwa	12,981	3628	(28)	368	(10)
Jamunaha	132,378	9642	(7)	366	(4)
Jhulaghat	1820	1799	(99)	107	(6)
Kakarbhitta	24,135	2864	(12)	129	(5)
Krishnanagar	19,572	946	(5)	128	(14)
Pashupatinagar	1528	75	(5)	2	(3)
Pulghat	973	466	(48)	1	(<1)
Rani	2450	969	(40)	86	(9)
Trinagar	66,927	27,817	(42)	2467	(9)
Bhittamod	2023	0	(0)	-	-

COVID-19= Corona Virus Disease-19; POE = Points of Entry.

**Table 2:** Socio-demographic and clinical characteristics of patients diagnosed positive for COVID-19 at 13 POE in Nepal: 19 March 2021–15 July 2021.

Characteristics of COVID-19 Patients		*n*	(%)
Total		3907	
Socio-demographic characteristics			
Age in years	1–5	21	(1)
	6–14	91	(2)
	15–29	2014	(52)
	30–44	1192	(30)
	45–59	438	(11)
	60 and above	151	(4)
Gender	Male	3331	(85)
	Female	576	(15)
Occupation	Unemployed	37	(1)
	Employed	129	(3)
	Migrant worker	3100	(79)
	Health worker	50	(1)
	Security person	28	(1)
	Student	255	(7)
	Business/Trade	13	(<1)
	Others *	20	(1)
	Not recorded	275	(7)
Nationality	Nepalese	3749	(96)
	Indian	158	(4)
Clinical characteristics			
Symptoms	Cough/Sore throat	214	(6)
	Fever	3693	(95)
	Other COVID-19 symptoms *	72	(2)

COVID-19 = Corona Virus Disease-19; POE = Points of Entry; Othetrs * = Housewife, Hotel Worker, Driver, Farmer; Other COVID-19 Symptoms = Symptoms other than Cough/Sore throat and Fever; Clinical = multiple response possible for symptoms.

**Table 3:** Types of isolation for COVID-19 patients at each POE: 19 March 2021–15 July 2021.

Points of Entry	Total Number Diagnosed with COVID-19	Home-Based Isolation *n* (%)		Community-Based Isolation *n* (%)		Hospital-Based Isolation *n* (%)		Returned to INDIA *n* (%)	
Total	3907	1261	(32)	2501	(64)	65	(2)	80	(2)
Belahiya	124	117	(94)	7	(6)	0	(0)	0	(0)
Gaddachauki	116	3	(3)	113	(97)	0	(0)	0	(0)
Gaur	13	0	(0)	13	(100)	0	(0)	0	(0)
Inarwa	368	271	(74)	15	(4)	2	(1)	80	(21)
Jamunaha	366	164	(45)	151	(41)	51	(14)	0	(0)
Jhulaghat	107	46	(43)	60	(56)	1	(1)	0	(0)
Kakarbhitta	129	126	(98)	0	(0)	3	(2)	0	(0)
Krishnanagar	128	120	(94)	0	(0)	8	(6.3)	0	(0)
Pashupatinagar	2	0	(0)	2	(100)	0	(0)	0	(0)
Pulghat	1	0	(0)	1	(100)	0	(0)	0	(0)
Rani	86	86	(100)	0	(0)	0	(0)	0	(0)
Trinagar	2467	328	(13)	2139	(87)	0	(0)	0	(0)
Bhittamod	0	0	(0)	0	(0)	0	(0)	0	(0)

COVID-19 = Corona Virus Disease-19; POE = Points of Entry.

## Data Availability

The data for this paper is available upon request from the principal investigator.

## Open Access Statement and Disclaimer

In accordance with WHO’s open-access publication policy for all work funded by WHO or authored/co-authored by WHO staff members, WHO retains the copyright of this publication through a Creative Commons Attribution IGO license (http://creativecommons.org/licenses/by/3.0/igo/legalcode, accessed on 7 June 2022) which permits unrestricted use, distribution and reproduction in any medium provided the original work is properly cited. There should be no suggestion that WHO endorses any specific organization, products or services. The views expressed in this article are those of the authors and do not necessarily reflect those of their affiliated institutions. The use of the WHO logo is not permitted. This notice should be preserved along with the article’s original URL.

## References

[1] World Health Organization Coronavirus (COVID-19) Dashboard. https://covid19.who.int/.

[2] Dickens B.L., Koo J.R., Tao Lim J., Sun H., Clapham H.E., Wilder-Smith A., Cook A.R. (2020). Strategies at Points of Entry to Reduce Importation Risk of COVID-19 Cases and Reopen Travel. J. Travel Med..

[3] World Health Organization (2021). Technical Considerations for Implementing a Risk-Based Approach to International Travel in the Context of COVID-19. Interim Guidance Annex to: Policy Considerations for Implementing.

[4] World Health Organization (2021). COVID-19 Strategic Preparedness and Response Plan.

[5] Mahmoudinobar F., Britton D., Montclare J.K. (2021). Protein-Based Lateral Flow Assays for COVID-19 Detection. Protein Eng. Des. Sel..

[6] Trombetta B.A., Kandigian S.E., Kitchen R.R., Grauwet K., Webb P.K., Miller G.A., Jennings C.G., Jain S., Miller S., Kuo Y. (2021). Evaluation of Serological Lateral Flow Assays for Severe Acute Respiratory Syndrome Coronavirus-2. BMC Infect. Dis..

[7] Peeling R.W., Heymann D.L., Teo Y.Y., Garcia P.J. (2022). Diagnostics for COVID-19: Moving from Pandemic Response to Control. Lancet.

[8] Bastola A., Sah R., Morales A.J.R., Lal B.K., Jha R., Ojha H.C., Shrestha B., Chu D.K.W., Poon L.L.M., Costello A. (2020). The First 2019 Novel Coronavirus Case in Nepal. Lancet Infect. Dis..

[9] Epidemiology and Disease Control Division, Nepal COVID-19 Statistics: Nepal. https://portal.edcd.gov.np/covid19/.

[10] Ministry of Health and Population (2022). Health Sector Response to COVID-19 Pandemic in Nepal.

[11] Ministry of Health and Population, Nepal Corona Virus Disease (COVID-19) Outbreak Updates and Resource Materials, Situation Report 399 and 691. https://heoc.mohp.gov.np/.

[12] Seddon D. India and Nepal in COVID-19 Crisis Together. https://www.nepalitimes.com/latest/india-and-nepal-in-Covid-19-crisis-together/.

[13] Central Bureau of Statistics (2012). Nepal Population and Housing Census 2011, National Report.

[14] Ministry of Health and Population (2020). Document on Standards for Health Examination Centre at Entry Point( Land Crossings).

[15] National Public Health Laboratory, Nepal List of Approved COVID-19 Antigen Kits. https://nphl.gov.np/covid19/antigen-kits/.

[16] Ministry of Health and Population (2020). Health Standard for Isolation of COVID-19 Cases.

[17] Lokossou V.K., Usman A.B., Sombie I., Paraiso M.N., Balogun M.S., Umeokonkwo C.D., Gatua J., Wagai J., Ouendo E.M., Nguku P. (2021). COVID-19 Pandemic in Economic Community of West African States (Ecowas) Region: Implication for Capacity Strengthening at Point of Entry. Pan Afr. Med. J..

[18] Meesit A., Kaewla W., Wiwanikit V. (2016). Forgotten Problems in Land Border Crossings. Ann. Trop. Med. Public Health..

[19] Rooij D.D., Belfroid E., Hadjichristodoulou C., Mouchtouri V.A., Raab J., Timen A. (2021). Assessing Training Needs in Infectious Disease Management at Major Ports, Airports and Ground-Crossings in Europe. BMC Public Health.

[20] Klinger C., Burns J., Movsisyan A., Biallas R., Norris S.L., Rabe J.E., Stratil J.M., Voss S., Wabnitz K., Rehfuess E.A. (2021). Unintended Health and Societal Consequences of International Travel Measures during the COVID-19 Pandemic: A Scoping Review. J. Travel Med..

[21] Wu Z., McGoogan J.M. (2020). Characteristics of and Important Lessons from the Coronavirus Disease 2019 (COVID-19) Outbreak in China: Summary of a Report of 72314 Cases from the Chinese Center for Disease Control and Prevention. JAMA J. Am. Med. Assoc..

[22] Richardson S., Hirsch J.S., Narasimhan M., Crawford J.M., McGinn T., Davidson K.W., Barnaby D.P., Becker L.B., Chelico J.D., Cohen S.L. (2020). Presenting Characteristics, Comorbidities, and Outcomes among 5700 Patients Hospitalized with COVID-19 in the New York City Area. JAMA J. Am. Med. Assoc..

[23] Koh H.K., Geller A.C., Vanderweele T.J. (2021). Deaths from COVID-19. JAMA J. Am. Med. Assoc..

[24] Elm E.V., Altman D.G., Egger M., Pocock S.J., Gøtzsche P.C., Vandenbroucke J.P. (2014). The Strengthening the Reporting of Observational Studies in Epidemiology (STROBE) Statement: Guidelines for Reporting Observational Studies. Int. J. Surg..

[25] Wirawan I.M.A., Sutarsa I.N., Astuti P.A.S. (2021). Healthy Tourism Initiative in the Age of COVID-19 in Indonesia. Asian Pac. J. Trop. Med..

[26] Yamano T., Pradhananga M., Schipani S., Samson J.N.G., Quiao L., Leuangkhamsing S., Maddawin A. (2020). The Impact of COVID-19 on Tourism Enterprises in the Lao People’s Democratic Republic: An initial Assessment.

[27] Abbas J., Mubeen R., Iorember P.T., Raza S., Mamirkulova G. (2021). Exploring the Impact of COVID-19 on Tourism: Transformational Potential and Implications for a Sustainable Recovery of the Travel and Leisure Industry. Curr. Res. Behav. Sci..

